# Cavernous sinus involvement in human papillomavirus associated oropharyngeal squamous cell carcinoma: case report of an atypical site of distant metastasis

**DOI:** 10.1186/s40463-018-0280-0

**Published:** 2018-05-09

**Authors:** David Forner, Derek Wilke, Matthew H. Rigby, Sidney Croul, Anuradha Mishra, Emad Massoud, David B. Clarke, Nathan Lamond

**Affiliations:** 1 Division of Otolaryngology – Head & Neck Surgery, Department of Surgery, Queen Elizabeth Health Science Center, Dalhousie University, Third Floor Dickson Building, Victoria General Site, 820 University Avenue, Halifax, B3H 1Y9 NS Canada; 2 Department of Radiation Oncology, Nova Scotia Cancer Centre, Dalhousie University, Dickson Building, Room 2200, main floor, 5820 University Avenue, Halifax, B3H 1V7 NS Canada; 3 Department of Pathology & Laboratory Medicine, Division of Anatomical Pathology, Dalhousie University, Room 635-B, 6th Floor, DJ Mackenzie Building, 5788 University Avenue, Halifax, B3H 2Y9 NS Canada; 4 Department of Ophthalmology & Visual Sciences, Dalhousie University, 1278 Tower Road, Room 2035, 2W Victoria, Halifax, B3H 2Y9 NS Canada; 5 Division of Neurosurgery, Department of Surgery, QEII Health Sciences Centre, Dalhousie University, 1796 Summer Street, Suite 3806, Halifax, B3H 3A7 NS Canada; 6 Division of Medical Oncology, Department of Medicine, Dalhousie University, QEII - Bethune Building, Suite 470 Bethune Building, 1276 South Park Street, Halifax, B3H 2Y9 NS Canada

**Keywords:** Human papillomavirus, Oropharyngeal squamous cell carcinoma, Cavernous sinus, Cranial nerve palsy

## Abstract

**Background:**

HPV-associated OSCC (HPV-OSCC) has been determined to be a distinct disease entity from non-HPV associated OSCC. Patients affected by HPV-OSCC generally have a more favourable prognosis, with improved rates of locoregional control and survival compared with their non-HPV counterparts. Despite this, HPV-OSCC has a similar rate of distant metastases. Interestingly, recent evidence has emerged that demonstrates more frequent atypical metastasis patterns when compared to non-HPV associated disease. To the best of our knowledge, this report describes the first case of a confirmed HPV-OSCC with distant metastasis to the cavernous sinus.

**Case Presentation:**

A 62-year-old non-smoking male presented to the head and neck oncology clinic with a five-month history of enlarging right neck mass causing neck pain, dysphagia, and dysphonia. HPV-associated base of tongue squamous cell carcinoma (cT4aN2c) was diagnosed, and he was treated with primary chemoradiation. Shortly after treatment, he presented with progressive bilateral cranial nerve palsies including left cranial nerve III and right cranial nerve VI involvement. Imaging identified masses in the left cavernous sinus with extension of tumor into the sella and in the right cavernous sinus at the level of Dorello’s canal. Endoscopic Image Guided Transsphenoidal biopsy of the left sellar mass confirmed distant metastases from the previously treated HPV-OSCC primary to the cavernous sinus. The patient was palliated with carboplatin and paclitaxel.

**Conclusion:**

The presented report is the first documented case of confirmed HPV-associated oropharyngeal squamous cell carcinoma metastasizing to the cavernous sinus, and the only HPV confirmed head and neck cancer case to present with metastasis to the cavernous sinus and limited extracranial disease. This case demonstrates the importance of recognizing presentations of atypical metastasis that are possible in HPV-associated oropharyngeal cancer. Given the rarity of metastasis to this region, vigilance in follow up is instrumental in early identification and treatment for these patients.

## Background

Oropharyngeal squamous cell carcinoma (OSCC) has traditionally been associated with tobacco and alcohol use. In the past, these exposures accounted for up to 75% of cases [1]. Public health initiatives have reduced smoking and alcohol consumption and resulted in a decline in head and neck cancers in general over the past three to four decades [2], yet rates of OSCC have continued to rise [3, 4]. The increased incidence of OSCC has been attributed to a growing proportion of diagnoses associated with infection by the human papillomavirus (HPV) [4]. This holds true in Canada as well, with an increase from 47% of oropharyngeal cancer cases being HPV-associated in 2000, to nearly 75% in 2012 [5].

HPV-associated OSCC (HPV-OSCC) has recently been recognized to be a distinct disease entity from OSCC occurring in the absence of HPV infection. Human papillomavirus association is most commonly identified with p16 immunohistochemistry, whereby HPV-OSCC cells display p16-positivity. In general, HPV-OSCC is associated with a more favourable prognosis when compared to OSCC occurring in the absence of HPV infection, with improved rates of locoregional control and patient survival following curative-intent treatment of localized disease [6]. Despite this more favourable prognosis, HPV-OSCC carries a similar risk of distant metastases of approximately 10% at three to five years [7, 8]. In addition, recent evidence demonstrates HPV-OSCC may also lead to atypical patterns of metastases. The most common sites of distant metastases in OSCC, regardless of HPV status, include lung, lymph nodes, bone and liver [9, 10]. However, HPV-OSCC has also resulted in atypical patterns, including dural metastasis [11], osseous metastases 11 years after initial treatment [12], and multiple brain metastases [13]. These findings are not limited to case reports, with retrospective reviews similarly suggesting that metastases amongst patients with HPV-related disease are more likely to include unusual sites [14]. Furthermore, studies examining long-term outcomes of distant metastases in HPV-OSCC have identified distinct “disseminating” phenotypes, with metastases to multiple sites, and “explosive” phenotypes, with complete organ involvement [15]. Finally, compared to non-HPV associated disease, patients with HPV-OSCC are more likely to experience late distant metastases, with disease appearing after two to three years [15].

To the best of our knowledge, this report describes the first case of a confirmed HPV-OSCC with distant metastasis to the cavernous sinus. We intend for this report to highlight the importance of recognizing presentations of atypical metastasis that are possible in HPV-associated oropharyngeal cancer.

## Case presentation

A 62-year-old male lifelong non-smoker was referred to the head and neck oncology clinic with a five-month history of enlarging right neck mass associated with neck pain, dysphagia, and dysphonia. He was otherwise healthy, aside from type II diabetes mellitus and a distant history of sarcoidosis. Physical examination, as well as contrast-enhanced CT scan of the neck, showed a large base of tongue mass that crossed the midline and projected into the suprahyoid epiglottis. There was a 4.6 × 3.4 cm mass representing a necrotic confluence of lymph nodes in right level IIA and IIB. Biopsy confirmed non-keratinizing, HPV-OSCC. Immunohistochemistry p16 testing was used for HPV diagnosis. PET/CT scan demonstrated bilateral level III lymphadenopathy, but did not show evidence of distant metastases. The patient was diagnosed with a cT4aN2c HPV-OSCC and referred to the institutional tumor board where a recommendation for definitive chemoradiation was made. Staging was according to the American Joint Committee on Cancer Seventh Edition. The patient received 70 Gy in 35 fractions to the primary site and involved nodes, with 56 Gy to the uninvolved regional nodes bilaterally, including the bilateral lateral retropharyngeal nodes. Due to the patient’s comorbidities the concurrent chemotherapy consisted of intravenous cetuximab given as a loading dose of 400 mg/m^2^ one week prior to the initiation of radiotherapy, followed by seven weekly doses of 250 mg/m^2^ concurrent with radiotherapy. During and soon after completing chemoradiotherapy, the patient experienced the expected toxicities and had evidence of favourable cancer response to treatment. A contrast enhanced CT scan of the neck performed five weeks after completion documented good anatomic response to treatment with resolution of all disease with the exception of a lymph node within right level II, which was much decreased in size, measuring 2 cm in greatest dimension.

**Fig. 1:**
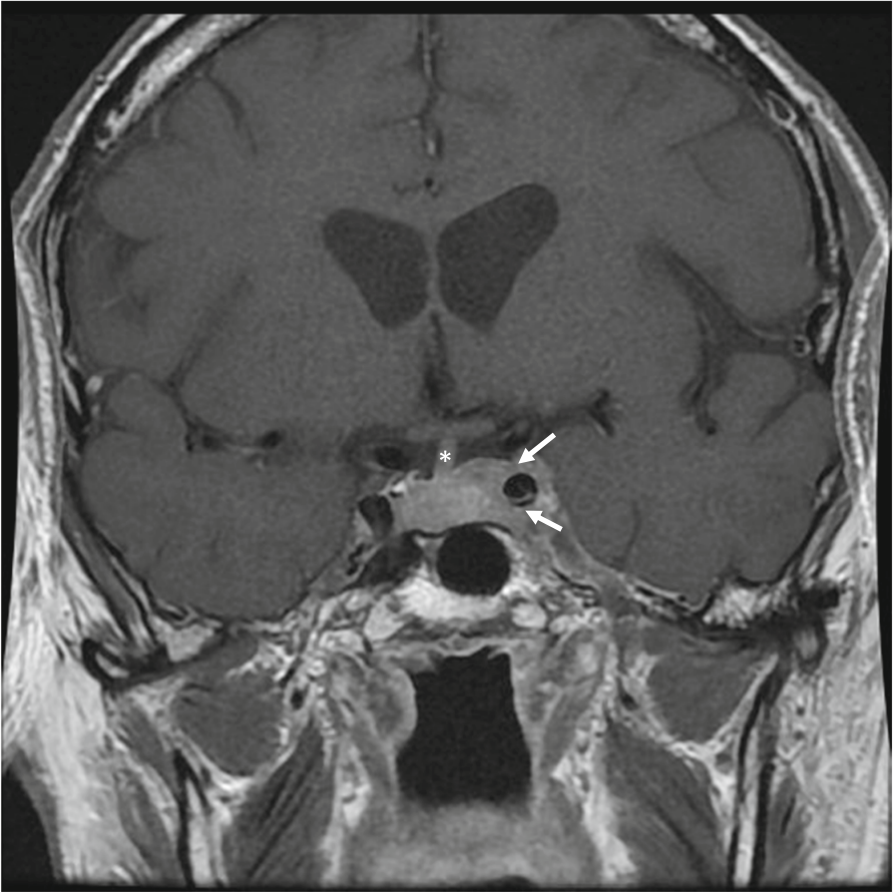
MRI scan of cavernous sinus mass. Coronal post-gadolium enhanced T1 MRI scan demonstrating soft tissue mass involving the left cavernous sinus and encasing the left internal carotid artery (arrows); note deviation of the hypothalamic-pituitary stalk to the right (*)

**Fig. 2:**
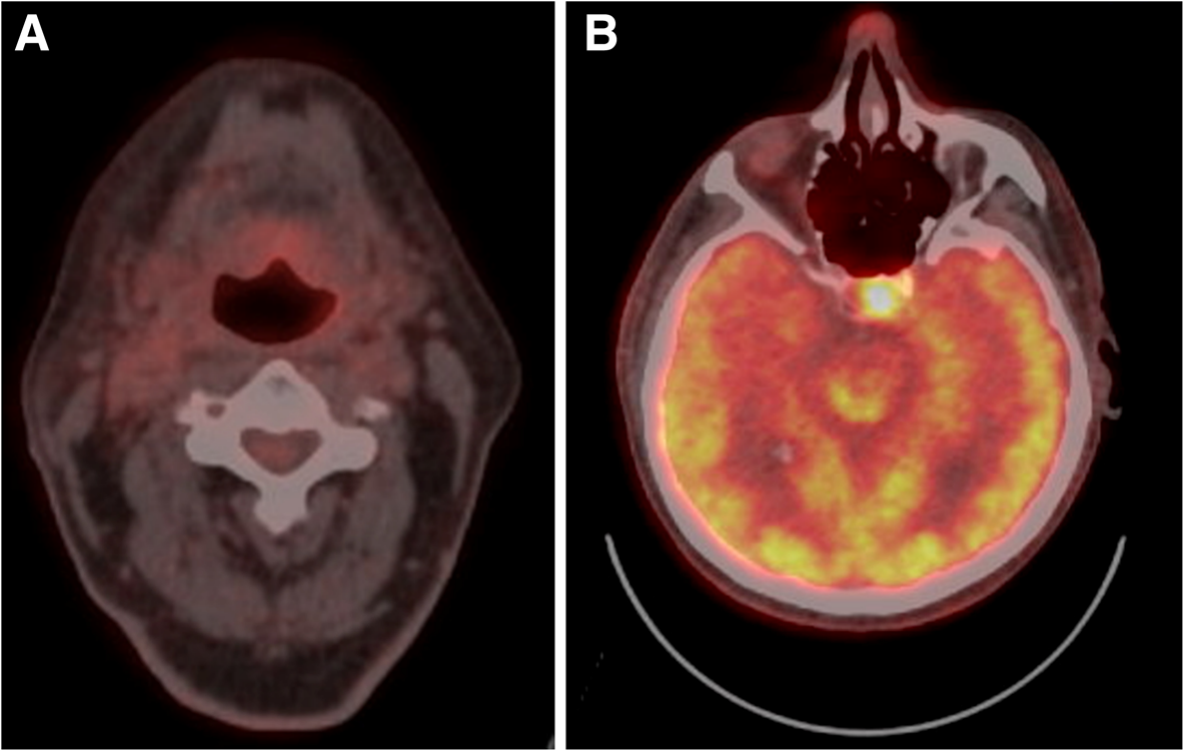
PET scan demonstrating resolution primary tumor and new sella lesion. PET demonstrating **a** metabolic resolution of the primary tumor and **b** intense focal FDG uptake within the left sella

Two months after completing chemoradiotherapy, the patient presented to medical attention with onset of binocular vertical diplopia. He was found to have subtle evidence of a new pupil-sparing, left-sided cranial nerve III palsy. His extraocular movement deficits progressed rapidly over one and a half weeks and upon repeat examination he was found to have a new right-sided cranial nerve VI palsy. Differential diagnosis included ischemia, neuromuscular disorder, metastasis, unrelated compressive lesion, and bilateral aneurysm. He was admitted to hospital for investigations. Electromyography showed no evidence of myasthenia or myopathy. Magnetic Resonance Imaging of the sella revealed two separate enhancing cavernous sinus lesions; one tumor in the left cavernous sinus that encased the internal carotid artery with extension into the sella (Fig. 1) and another involving the right cavernous sinus and clivus at the level of Dorello’s canal. PET/CT scan demonstrated resolution of his original primary tumor and cervical lymphadenopathy, suggesting complete metabolic response within the chemoradiotherapy field (Fig. 2a). However, intense focal FDG uptake was demonstrated within the aforementioned two cavernous sinus lesions (Fig. 2b) as well as FDG uptake within a new 1 cm hypodensity within segment 4A of the liver.

**Fig. 3:**
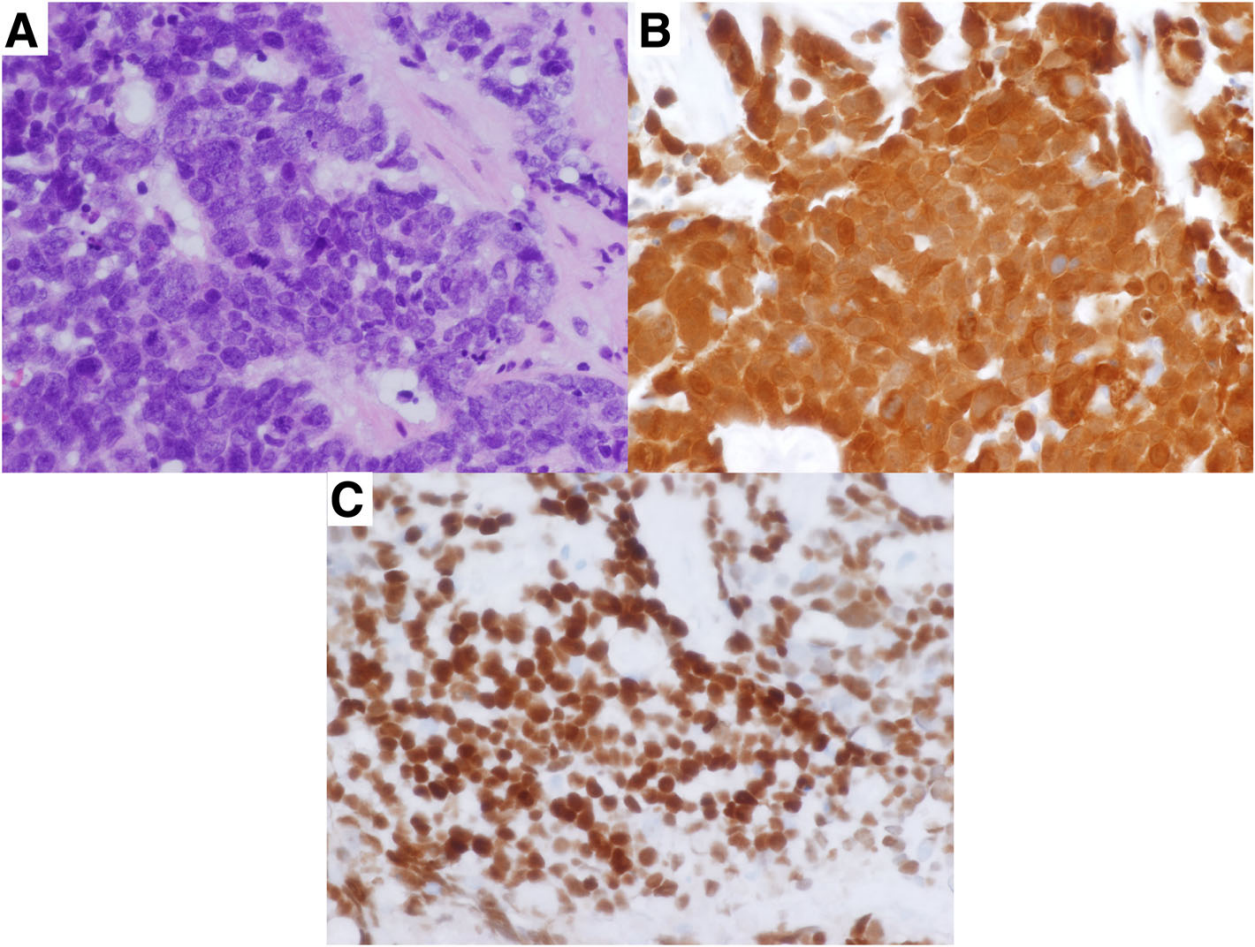
Histology sections of sellar lesion. Biopsy of sellar tumor demonstrating non-keratinizing p16 squamous cell carcinoma. Immunohistochemistry demonstrating: **a** poorly differentiated cancer with squamous differentiation; **b** immunohistochemistry demonstrating diffuse p16 positivity; and **c** diffuse p63 positivity

The patient underwent Endoscopic Image Guided Transsphenoidal biopsy of the sellar tumor which showed a malignant basaloid tumor consistent with non-keratinizing p16-positive squamous cell carcinoma, likely of oropharyngeal origin (Fig. 3) and consistent with early distant metastases from his HPV-OSCC. Discussion with both radiation oncology and medical oncology was held, outlining the poor prognosis associated with metastatic head and neck cancer. The patient began palliative chemotherapy with carboplatin and paclitaxel, and has tolerated this well, with mild improvement in his symptoms in the form of improved extraocular eye movement and return of left eye vision.

## Discussion

Metastases involving the cavernous sinus are rare and have previously been described in several solid tumors. Cancers with primary tumors outside of the head and neck have been shown to occasionally metastasize to the cavernous sinus, including carcinomas of the lung [16], colon [17, 18], and liver [19]. Head and neck cancers have also been described to metastasize to the cavernous sinus, including squamous cell carcinomas with laryngeal primaries [20], papillary thyroid cancer [21], and facial tumors [22, 23].

Two cases of oropharyngeal squamous cell carcinoma with metastasis to the cavernous sinus have been recently described. In one, disease progression was advanced on initial presentation, with a relatively small primary tumor but substantial nodal involvement and lung metastases (cT1N2bM1). In the other, nodal involvement was also extensive on the patient’s initial presentation, with N2c disease. There were no additional sites of metastasis in that case. However, no HPV testing or smoking histories were reported in either case, calling into question the nature of the cancer [24, 25]. Wirth and colleagues [26] highlighted an HPV confirmed squamous cell carcinoma of oral cavity origin with post-treatment metastasis to the cavernous sinus presenting as diplopia, ptosis, and ophthalmoplegia. In that case, the tumor involvement was extensive (pT4N2c disease) with surgical and adjuvant chemoradiation treatments. However, the association of HPV and oral cavity cancer is under debate, and it remains unclear whether HPV-associated oral cavity cancer should be considered a distinct disease entity from non-HPV associated disease as is considered the case for oropharyngeal cancer [27]. The currently presented case is therefore the first documented report of confirmed HPV-OSCC to the cavernous sinus.

HPV-associated oropharyngeal cancer is considered to be a distinct disease entity and therefore has a different natural history and prognosis compared to non-HPV associated oropharyngeal cancer. Locoregional control and overall survival of HPV patients is better, with long term survival approaching an improvement of over 40%. However, a subset of patients develop early, widespread metastases and fair very poorly [6]. As well, rare, atypical sites of distant metastasis are more common in HPV-associated disease. As was the case in our patient, extensive nodal disease on presentation is common in HPV-associated oropharyngeal cancer, and has been determined to be a predictor of poor outcomes and shorter disease-free survival [28]. Extensive smoking history has also been proposed as a predictor of poor outcomes in HPV-OSCC [7]. The patient in the current report was a lifetime non-smoker, and unfortunately developed distant metastasis. This highlights that although our ability to predict disease outcome is improving [7, 28, 29], work still remains to determine which patients may suffer from these early metastases.

Although outcomes differ, treatment of HPV-OSCC currently aligns with that of non-HPV OSCC and may include surgical resection, radiation therapy alone, or chemoradiation. Among head and neck cancer populations unselected for HPV status, the evidence supporting the addition of concurrent chemotherapy using either cisplatin or cetuximab has shown definitive improvements in overall survival and locoregional control rates compared with definitive radiation alone [30, 31]. However, the rate of distant metastases was not significantly improved by concurrent treatment in these studies. Recent studies also suggest improved outcomes and overall survival with the addition of concurrent chemotherapy using either cisplatin or cetuximab to radiation therapy in locoregionally advanced HPV-OSCC, specifically [28, 32] . While high-dose cisplatin may result in improved distant metastases rate in HPV-OSCC [28], there remain no data to suggest that concurrent cetuximab results in significant improvement in distant metastases rate over definitive radiotherapy alone. Despite the addition of concurrent cetuximab in the patient presented here, distant metastasis occurred rapidly.

The cavernous sinuses are paired dural venous sinuses that contain, either running directly through or within the lateral wall, cranial nerves III, IV, V_1_, V_2_, and VI, as well as the internal carotid artery. Due to these anatomic relationships, lesions of the cavernous sinus may present with headache, chemosis, ophthalmoplegia, proptosis, miosis, diplopia, or sensory deficits in the ophthalmic and maxillary nerve distributions. Indeed, cavernous sinus metastases are capable of presenting with these signs and symptoms, and recognition of this pattern is necessary in HPV-OSCC patients as this disease has shown a propensity for atypical metastases. Metastasis to this region is rare, and having two separate metastatic tumors of the cavernous sinuses become simultaneously symptomatic was indeed extraordinary. Vigilance in close follow up of newly symptomatic patients is instrumental for early identification of tumor metastasis and for treating these patients.

## Conclusion

The presented report is the first documented case of confirmed HPV-associated oropharyngeal squamous cell carcinoma metastasizing to the cavernous sinus, and the only HPV confirmed head and neck cancer case to present with cavernous sinus involvement in the absence of widespread metastases. This case demonstrates the importance of recognizing presentations of atypical metastasis that are possible in HPV-associated oropharyngeal cancer.

## Abbreviations

CT: Computed tomography
HPV: Human papillomavirus
HPV-OSCC: Human papillomavirus associated oropharyngeal squamous cell carcinoma
OSCC: Oropharyngeal squamous cell carcinoma
PET: Positron emission tomography
